# Onchocerciasis
**Reports to the Local Government Board on Public Health and Medical Subjects.* New series, No. 45. Reports by Dr. A. W. J. MacFadden and Dr. Robert T. Leiper, F.Z.S., on a parasitic condition (*onchocerciasis*) met with in Australian beef. Food reports, No. 11. (London: Darling and Son, Ltd. 1911. Price 6d.)


**Published:** 1911-05-13

**Authors:** 


### SPECIAL ARTICLE.

### ONCHOCERCIASIS.*

Early in 1910 the attention of the Local Government Board was drawn to the presence of certain nodular masses in frozen quarters of Australian meat. On an inquiry made on behalf of the Board these nodules were found to contain a parasitic worm formerly called *spiroptera reticulata*, and known to occur in Australian cattle. The first part of the present report is by Dr. Macfadden and deals with the inspection of this meat at English ports. The nodules were at first thought to be merely a surface affection of the flank and brisket of the forequarter and consequently easily capable of removal, but later they were found to be also more deeply situated, especially in the intermuscular fat, as well as in the hindquarter near the knee-joint. Rigid examination of a large number of quarters made it clear that if freedom from parasitic nodules were to be secured it would be necessary either to examine in detail every forequarter or else to cut off from it those parts, namely, the flanks and briskets, to which they were known to be chiefly confined. The first course being impracticable, the second was decided on. The meat was kept in cold store until all the flanks and briskets had been sawn off, and the latter alone retained for further examination subject to the wish of the importer to utilise them further. If notice was given that they were required for industrial purposes they were allowed to be taken away after being rendered unfit for food. If, on the other hand, they were to be used for food purposes they were thawed out and cut up for examination by the officers in authority. Since hindquarters were only affected round the stifle-joint it was only necessary to make an incision into it while frozen to pass or reject the piece. In practice few of the importers have desired to make use of their flanks and briskets for food purposes, but have surrendered them to the sanitary authorities for destruction. Part II. consists of a report on the structure and bionomic characters of the parasite by Dr. Leiper. He considers that it should be included in the genus *Onchocerca*, and not in that of the *Spiroptera*, and he proposes to call the condition to which it gives rise "Onchocerciasis." It was first observed by Morris of Sydney in 1880, and since then has been recognised by other observers in different parts of the Australian continent. It has been noticed also in cattle coming from the United States of America, the Malay States, Java, and India. The parasites are long thread-like worms, male and female, the former occupying the centre, the latter the periphery of the nodule. The female is the larger and shows to the naked eye a faint but regular striation. The male is recognised by its slender form, its apparent lack of striation and its loose attachment to the stroma of the nodule. The author gives a full description of the anatomical characteristics of each sex into which it is unnecessary to follow him here. So far as is known no parasitic worm can develop to sexual maturity within the body of the host in which it received its birth without first passing a more or less brief period of its life outside. Nothing at present is known of this extra-corporeal life so far as the *Onchocerca* is concerned, nor of its mode of entry or exit from the host, yet on analogy with other members of the family Filariidæ, it must take up its abode in the body of some arthropod, probably a biting insect. Thus infection of man from eating food containing these young in a living state is impossible. It should not be difficult to identify this insect which acts as intermediate host, and so by appropriate prophylactic measures to eradicate the disease or at any rate diminish it in cattle. The worms and their young do not appear capable of surviving for more than a few hours the death of the cattle. No evidence of vitality of the worm or its embryo has been met with in the case of Australian beef reaching this country. The author does not agree with Dr. Cleland and Dr. Johnstone that "from the butcher's standpoint they (the nodules) may be viewed as interfering with the general appearance of the affected part," or that from the point of view of the general public "they may be looked on as pathological curiosities." The nodules are the product of changes taking place in the tissues as a result of some acrid toxin excreted by the worms, and in his opinion their presence in meat intended for human consumption is undesirable for that reason. The pamphlet is brought to a close by a table of references and three plates, two of which are drawings made of the worms as seen under the microscope, and the third is from a photograph of a piece of beef in which an unusually large number of nodules is present.

* *Reports to the Local Government Board on Public Health and Medical Subjects.* New series, No. 45. Reports by Dr. A. W. J. MacFadden and Dr. Robert T. Leiper, F.Z.S., on a parasitic condition (*onchocerciasis*) met with in Australian beef. Food reports, No. 11. (London: Darling and Son, Ltd. 1911. Price 6d.)

